# Predicting treatment response to IL6R blockers in rheumatoid arthritis

**DOI:** 10.1093/rheumatology/keaa529

**Published:** 2020-08-31

**Authors:** Bako Nouri, Nisha Nair, Anne Barton

**Affiliations:** k1 Centre of Genetics and Genomics Versus Arthritis, Centre for Musculoskeletal Research, University of Manchester; k2 NIHR Manchester Musculoskeletal BRU, Central Manchester Foundation Trust, Manchester Academic Health Sciences Centre, Manchester, UK

**Keywords:** IL6R blockers, rheumatoid arthritis, treatment response

## Abstract

Patients with severe, active RA who have not responded to conventional therapy may receive biological disease modifying anti-rheumatic drugs (bDMARDs). However, 40% of cases do not achieve complete disease control, resulting in a negative impact on patient quality of life and representing a waste of healthcare resources. Ongoing research seeks to establish biomarkers, which can be used to predict treatment response to biologics in RA to enable more targeted approaches to treatment. However, much of the work has focused on one class of biologic drug, the TNF inhibitors (TNFi). Here, we will review the current state of research to identify biomarkers predictive of response to the class of bDMARDs targeting the IL6R. While success has been limited thus far, serum drug and low ICAM1 levels have shown promise, with associations reported in independent studies. The challenges faced by researchers and lessons learned from studies of TNFi will be discussed.

**Rheumatology key messages**

- Many studies have investigated biomarkers to predict response to IL6i with limited success so far.
- Serum drug and low ICAM1 levels have been correlated with better response in independent studies.
- Study designs require optimization and multi-biomarker panels may be required to capture variability in response.

## Introduction 

RA is a chronic inflammatory disease of synovial joints, which, if left untreated causes severe pain, functional disability and joint damage [1]. Guidelines recommend early and effective control of disease activity as poor control in early disease is reported to correlate with poorer long-term outcomes including joint damage and disability [2]. Initially, conventional synthetic disease modifying anti-rheumatic drugs (csDMARDs) such as methotrexate are recommended as first-line treatment [3]. However, patients with severe, active RA who have not responded to conventional therapy may receive biological disease modifying anti-rheumatic drugs (bDMARDs). Since their introduction as therapy for RA, bDMARDs have significantly improved the quality of life for patients and increased their chances of experiencing disease remission [4]. A range of bDMARDs target different pathways of the inflammatory process driving RA pathogenesis. These include drugs inhibiting the TNF, IL-6, T-cell co-stimulatory and B-cell pathways, and JAK/STAT signalling. However, for each drug class, clinical trials show that the treatments fail to achieve good disease control in ∼40% patients, but, at present, there is no scientifically-driven strategy for selecting which bDMARDs a patient should receive; guidelines in several countries recommend selection on the basis of cost, using the cheapest drug first [5]. Failure to respond to a bDMARD has a negative impact on both the patient and the economy. For example, while patients are receiving ineffective treatments, they will have ongoing symptoms, be exposed to the potential adverse effects of the treatment, and be at risk of joint damage while the administration of ineffective treatments represents a waste of healthcare resources [6].

As a result, numerous studies have been conducted with the aim of establishing biomarkers, which can be used to reliably predict treatment response to bDMARDs in order to allow informed treatment selections. Much of this work has focused on predicting response to TNFi [7]. Clinical features, drug adherence, genetic variants, transcriptomic factors and proteomic measures including autoantibodies, drug levels and anti-drug antibody levels have all been studied as potential biomarkers to predict treatment response. Far fewer studies have explored biomarkers of response to other classes of bDMARDs but here we focus on those that have investigated potential biomarkers of response to drugs targeting the IL-6 pathway.

## IL-6 pathway inhibitors

IL-6 is a pivotal cytokine in mediating inflammation and systemic features of RA; these include synovitis, fatigue, anaemia, anorexia and bone loss [8]. The biological activity of IL-6 is mediated by a receptor complex composed of two distinct membrane-bound glycoproteins, an 80 kDa IL-6 receptor (IL-6R) and a 130 kDa signal-transducing element [Glycoprotein 130 (gp130)] [9]. A soluble form of the IL-6R (sIL-6R) can also bind IL-6 with a similar affinity as the membrane bound IL-6R, forming a complex that triggers dimerization of gp130 and induces responses on cells that do not express the membrane-bound IL-6R [10]. This process is referred to as trans-signalling. Soluble Glycoprotein 130 (Sgp130) is the naturally occurring antagonist of the IL-6/sIL-6R complex that selectively inhibits IL-6 signalling, and is secreted when the gene gp130 is spliced. 

Due to the important role of IL-6 in mediating inflammation, IL-6 inhibitors (IL6i) have been developed and, currently, there are two licensed for the treatment of RA; tocilizumab (TCZ) and sarilumab [11, 12]. Both the humanized monoclonal antibody (mAb) TCZ and the fully human mAb sarilumab target the soluble and membrane-bound IL6R, though TCZ has a 15 to 22-fold weaker binding affinity [13]. A recent study found that the stronger binding affinity of sarilumab manifested as a higher receptor occupancy and greater reduction in CRP levels compared with TCZ [14]. Although they are usually prescribed when there has been an inadequate response to one or more bDMARDs, they are also licensed to be used as the first-line biologic [3]. Many studies have reported the efficacy of TCZ [15, 16] and sarilumab [17, 18] for the treatment of RA, with relatively good response rates observed for both drugs. For TCZ, several studies have demonstrated equal effectiveness as a monotherapy as when given in combination with methotrexate [19–21] and in both seropositive and seronegative subgroups [22]. The drugs differ in their frequency of administration with TCZ administered weekly while sarilumab is administered every two weeks. Of the two, TCZ was licensed and came to market first and so much of the research investigating potential biomarkers of response to drugs targeting the IL-6 pathway has focused on TCZ.

## Predicting treatment response to tocilizumab

Many studies have investigated the relationship between different biomarkers and clinical response to TCZ. In some cases, the features selected for investigation were based on findings on predictors of response to TNFi and, where findings are replicated, suggest that the predictive feature is a prognostic biomarker rather than specific to a particular treatment. The biomarkers reported to correlate with response to TCZ are summarized in Table 1 and include clinical, genetic, transcriptomic and serum biomarkers.

**Table 1:** Biomarkers reported to be associated with treatment response to tocilizumab

Biomarker category	Biomarker reported to predict treatment response to TCZ	Sample size	Study	*P*-value for association
Clinical	Baseline CRP	204 126	Pers *et al.*, 2014 [23] Narváez *et al.*, 2016 [24]	0.022 0.027
	Baseline ESR	126	Narváez *et al.*, 2016 [24]	0.003
Genetic	IL6R SNPs (rs12083537, rs2228145, rs4329505)	79	Enevold *et al.*, 2014 [25]	0.00004
	GALNT18 C-allele CD69 A-allele	79	Maldonado-Montoro *et al.*, 2016 [26]	0.02 0.023
Transcriptomic	Type 1 IFN response genes (IFI6, MX2, OASL) MTG1	40	Sanayama *et al.*, 2014 [27]	0.038, 0.012, 0.038 respectively 0.003
Serum	Serum D-dimer level Serum IL-1ß level	65	Okano *et al.*, 2016 [28]	0.005 <0.002
	Serum 14-3-3η level	149	Hirata *et al.*, 2015 [29]	0.0014
	Baseline Haemoglobin level	126	Narváez *et al.*, 2016 [24]	0.02
	Serum gp130 level logIL-6 logIL-8 logEotaxin logIP-10 logVEGF logsTNFR-I logsTNFR-II logGM-CSF	138	Kazuko *et al.*, 2015 [30]	0.002 0.002 <0.0001 <0.0001 0.002 0.039 <0.0001 0.03 0.0003
	sICAM1^low^/CXCL13^high^	198	Dennis *et al.*, 2014 [31]	0.004
	sIL-6R levels	43	Nishina *et al.*, 2013 [32]	0.02
	RF Positivity	6 studies (meta-analysis)	Maneiro *et al.*, 2013 [33]	N/A
	TCZ drug levels	100 126	Arad *et al.*, 2019 [34] Benucci *et al.*, 2016 [35]	0.001 0.0005
*Cellular*	NK cells	92 (20 received TCZ)	Daïen *et al.*, 2016 [36]	0.01

CD69:  cluster of differentiation 69; CXCL13:  C-X-C motif chemokine ligand 13; GALNT18:  polypeptide N-acetylgalactosaminyltransferase 18; ICAM1:  intracellular adhesion molecule 1; IFI6:  IFN alpha-inducible protein 6; MTG1:  mitochondrial ribosome associated GTPase 1; MX2:  MX dynamin like GTPase 2; OASL: 2'-5'-oligoadenylate synthetase-like; SNP: single nucleotide polymorphism; TNFR: TNF receptor.

## Clinical biomarkers

Studies of response to TNFi have consistently identified that clinical features correlate with response to therapy. Factors associated with a higher likelihood of response include obesity, higher pre-treatment disease activity and higher functional ability [37, 38]. Similar findings have been reported for IL6i; for example, two studies have reported that features of high pre-treatment disease activity, such as high baseline ESR, high baseline CRP and higher baseline DAS28-ESR scores also associate with response to TCZ [23, 24]. This is expected as patients with higher baseline disease activity scores have more ‘room to improve’ compared with patients with a moderate baseline disease activity (regression to the mean). Pers *et al.* showed that younger age also correlated with better treatment response [23]. Previous work has also shown that BMI correlates with subsequent treatment response to TNFi [38], but studies of TCZ response have been conflicting with two finding no relationship [39, 40] while a more recent study of a smaller cohort reported an inverse association of BMI with clinical response [34].

## Genetic biomarkers

Single nucleotide polymorphisms (SNPs) refer to loci with alleles that differ by a single nucleotide, with the less common allele present at a level of at least 1% in the population [41]. A genetic polymorphism within the gene encoding IL6R has been confirmed to be associated with susceptibility to RA [42]. Because the gene is the target of TCZ, several studies have investigated whether the same or other variants across the gene are associated with response to TCZ therapy. Although a larger cohort of 927 patients found no association, [43], a study of 79 patients reported that a haplotype of variants encompassing three SNPs associated with less improvement in the swollen joint count (SJC) scores between baseline and 6 months [25]. Due to the small sample size and conflicting findings, larger studies are necessary to resolve whether the genetic variation impacts therapeutic response.

Rather than targeting the *IL6R* as a candidate gene, Wang *et al.* adopted a hypothesis-free genome wide association study (GWAS) approach in a cohort of 1683 subjects and reported associations between eight novel loci and response to TCZ treatment and these are displayed in Table 2 [44]. The correlation between the SNPs related to CD69 and GALNT18 and response to TCZ were validated in a small candidate gene study of 79 patients [26]. These findings require replication in independent, large data sets before having confidence that they represent reliable biomarkers and, alone, they are unlikely to be clinically useful as they capture only a small amount of the variance in response. However, if confirmed in larger cohorts, they may prove useful in an algorithm combining clinical, genetic and other features to predict response.

**Table 2:** Eight loci associated with TCZ response [44]

SNP variant markers that achieved confirmation	Minor allele frequency	Coding	ß/OR	*P*-value
rs11052877	0.38	*CD69*	0.56	0.0039
rs4910008	0.47	*GALNTL4*	−3.28	0.0063
rs9594987	0.44	*ENOX1*	−0.1	0.016
rs10108210	0.41	—	0.09	0.028
rs703927	0.48	—	0.68	0.022
rs703505	0.42	*KCNIP1*	−0.09	0.031
rs1560011	0.42	*CLEC2D*	0.72	0.046
rs7055107	0.48	*SLC9A7*	−0.28 & −0.21	0.006 & 0.05

CD69: cluster of differentiation 69; CLEC2D: c-type lectin domain family 2 member D; ENOX1: ecto-NOX disulphide-thiol exchanger 1;GALNTL4: polypeptide N-acetylgalactosaminyltransferase 4; KCNIP1: kv channel-interacting protein 1; OR: odds ratio; SLC9A7: solute carrier family member A7; SNP: single nucleotide polymorphism.

## Transcriptomic biomarkers

The transcriptome refers to the messenger RNA or amount of gene product expressed in a cell or tissue at a specific time point [45]. Several studies have reported transcriptomic biomarkers to be associated with TCZ treatment response. A small study of 40 patients reported a significant difference between TCZ responders and non-responders in their relative expression of Type 1 IFN response genes (*IFI6, MX2* and *OASL*) and Metallothionein 1 G (*MT1G*) genes in peripheral blood mononuclear cells. This finding was replicated in a validation cohort of 20 patients [27]. While further replication is required in independent datasets, these findings provide promise that transcriptomic signatures of response may be identified. Some researchers argue that the site of joint inflammation, the synovial tissue itself, may be more informative but no studies to date have reported association of tissue markers with IL6i response.

## Serum biomarkers

Serum biomarkers refer to proteins that are measured in the patient’s serum and many studies have investigated these in relation to predicting treatment response to TCZ. For example, in a cohort of 65 patients, low serum D-dimer and IL-1β levels at 4 weeks were reported to predict treatment response to TCZ at 52 weeks [28]. In a Japanese cohort, pre-treatment serum 14–3-3η levels predicted 1-year DAS28 remission in patients treated with TCZ [29] while a different study of 126 patients found baseline haemoglobin concentration to correlate with treatment response at 3 months [24]. Another study found that among TCZ-treated patients with serum gp130 levels above 0.2 μg/ml, ∼60% were in remission compared with only 19% where levels were below that value [30]. The same study reported logIL-6, logIL-8, logEotaxin, logIP-10, logVEGF, logsTNFR-I and logsTNFR-II pre-treatment serum levels were predictive of treatment response at 16 weeks in ‘naïve’ TCZ patients, referring to patients taking bDMARDs for the first time, while logGM-CSF and logIP-10 were predictive in ‘non-naïve’ patients, referring to patients who had failed to respond to between one and three prior bDMARDs. Finally, analysis of serum samples from the Phase 4 ADACTA study comparing TCZ monotherapy with adalimumab monotherapy reported that both serum ICAM1 and CXCL13 were associated with response to TCZ by week 24 [31]. Specifically, patients with low ICAM1 and high CXCL13 levels showed the greatest response to TCZ, with 49% achieving an ACR50 response (*P* =0.004) while 45% achieved an ACR70 response (*P* =0.004), though this association requires replication.

Baseline levels of soluble IL-6-receptor (sIL-6R) were reported to predict clinical remission in TCZ-treated patients in a relatively small study of 43 patients by Nishina *et al.* [32]. While these reports are promising, without replication in independent large sample sets, confidence that any represent consistent and reliable biomarkers of response is currently limited.

Autoantibodies have been found to associate with response to both TNFi and RTX [33, 46, 47]; therefore, they have also been investigated for association with TCZ response and a meta-analysis published in 2013 found that RF positivity at baseline predicted better response to TCZ [33]. However, several individual studies have reported no association between RF positivity and response [48, 49], and so the association at present is not convincing enough to warrant its use in clinical practice.

In studies of TNFi, drug levels have been consistently reported to correlate with subsequent treatment response across a range of different subclasses [50, 51]. The presence of anti-drug antibodies inversely correlates with drug levels but the latter shows higher correlation with subsequent response. While retrospective analyses of TNFi-treated cohorts suggest that routine drug monitoring in clinical practice may be cost-effective, few prospective studies have been performed and a recent review by NICE found there was insufficient evidence on which to make recommendations [52]. Clearly, however, this is an area of active research interest and two studies have investigated the relationship between serum drug levels of TCZ and treatment response. The most recent study found, using a multivariate binary generalized estimating equation (GEE) model, every increase of 10 µg/ml in TCZ concentration was associated with being in a state of CDAI remission or low disease activity with an odds ratio of 1.41, *P*=0.001 [34]. An earlier study compared disease activity at 6 months of patients with TCZ drug concentration <10 µg/ml and >10 µg/ml, reporting significantly different mean DAS28 scores of 3.09 and 2.78, respectively (*P* =0.0005) [35].

## Cellular studies

Very few studies have investigated cell subtypes as predictors of response to treatment in RA. One that did reported that a higher baseline NK cell count was associated with clinical remission after 3 months of treatment with TCZ [36], but no association was observed with response to TNF inhibitors. Replication is required in a larger cohort but the results suggest that measurement of NK cells at baseline could predict response to TCZ.

## Predicting treatment response to sarilumab

At present, only two studies have been conducted to investigate potential biomarkers that can predict response to sarilumab in patients with RA. A study of 291 patients found that low baseline levels of sICAM-1 predicted DAS28-CRP and CDAI low disease activity response after 12 weeks (*P*=0.0332 and 0.0346 respectively), in keeping with similar findings for TCZ [31, 53]. Elevated baseline levels of IL-6 were also reported to correlate with a greater response to sarilumab in a large cohort of 1193 patients [54]. Despite the positive findings and the relatively large sample sizes in both studies, the findings still require replication in independent cohorts.

## Considerations for future studies

While several biomarkers have been reported to correlate with treatment response to IL6i in the literature, generally these studies have been undertaken in small sample sizes and there is a lack of replication, so findings are inconclusive. Studies of TCZ suggest that drug levels are potential biomarkers of future response as findings have been consistent. Furthermore, independent studies in TCZ- and sarilumab-treated patients have both reported association of low baseline sICAM1 with treatment response, again providing encouragement that reliable serum biomarkers can be identified. However, other potential biomarkers still require further validation in larger, independent cohorts before being assessed for their utility in clinical practice.

Although studies may identify biomarkers that correlate with treatment response, these biomarkers may not be useful in clinical practice as they may not explain large amounts of the variance in response alone to be clinically useful. As a result, there is growing advocacy for the implementation of a multi-biomarker algorithm that utilizes data from multiple different clinical, environmental and biological measures to predict treatment response. Although there is limited research in this particular area at present, a study published by Tasaki *et al.*in 2018 highlighted the usefulness of a multi-omics approach to understanding treatment response in RA [55]. The authors monitored treatment response to TCZ, infliximab and methotrexate at the transcriptome, proteome and immunophenotype level, reporting that patients who achieved molecular remission across multiple data types were more likely to experience better long-term outcomes, compared to those patients who achieved remission according to a single data type. Further studies are required to investigate the feasibility and utility of a multi-omics and combined modelling approach to predicting treatment response.

## Assessment of treatment response

An important issue for researchers seeking to identify biomarkers to predict treatment response is the actual measurement of response. The Disease Activity Score 28 (DAS-28) is the most commonly used measure of disease activity in RA, and combines the number of tender and swollen joints from 28 assessed joints, a patient-reported visual analogue score of global well-being and inflammatory markers such as CRP and ESR in an algorithm to calculate a composite score of disease activity. It is worth noting that in patients receiving IL6Ri, the DAS-28 is confounded because CRP levels rapidly normalize regardless of actual clinical efficacy, and so DAS-28 is not a reliable measure of treatment response. Instead the Clinical Disease Activity Index (CDAI) is used, which combines four parameters; the tender and swollen joint count; and measures of patient and physician global assessment of disease activity. In both measures of disease activity, subjective components are heavily weighted. Previous studies of the DAS28 subcomponent changes with response to TNFi have shown that the subjective components are more correlated with psychological factors [56]; only the swollen joint count and ESR/CRP changes show heritability [57] and the same two factors are the ones that correlate best with ultrasound measures of synovial inflammation [58]. Given that CDAI incorporates even fewer objective measures than DAS28, how it performs as a measure of treatment response in observational studies requires consideration. If an outcome is inaccurately measured, identifying predictors of that outcome becomes even more challenging. Future work may require identifying more objective measures of disease activity for real-world studies to ensure consistency of measurement.

## Effect of adherence

Another significant issue in studies investigating potential biomarkers is that patient adherence is rarely accounted for, but non-adherence is common and correlated with non-response. For example, of 392 patients receiving biological therapies, 27% reported non-adherence at least once by 6 months and non-adherence was associated with subsequent non-response by 12 months [59]. Therefore, patients who may have responded (based on the presence of a biomarker) can be misclassified as non-responders due to non-adherence to treatment. The failure to either ensure study participants are adherent or make necessary adjustments based on adherence significantly reduces the accuracy of predictive biomarker studies and future studies should consider ways to mitigate this common and important confounder.

## Conclusion

In conclusion, there have been a variety of studies conducted aiming to identify biomarkers that can predict treatment response to IL6i, particularly TCZ, in patients with RA. Despite the substantial investment, there has been limited success thus far and no biomarker has yet shown to be clinically useful in this setting. However, there are some promising findings that merit replication studies in independent and larger cohorts, with drug levels and serum sICAM1 levels showing the greatest promise so far. In addition, issues affecting the reliability of studies need to be addressed, such as confounding and the use of subjective measures of disease activity. The early findings from biomarker studies suggest that biomarkers considered individually are unlikely to be useful in clinical practice and a multi-biomarker prediction model may provide the most utility.
